# Quintuplets

**Published:** 1917-06

**Authors:** 


**Quintuplets.** W. Martin, Brit. Med. Jour., Mch. 17, reports a case, 4 boys and 1 girl, living ½-28 hours. The mother was 39 years old, 8-para, all previous children living, herself one of eight children, no family history of multiple births except that a sister had had twins once.

